# Knowledge evaluation in dementia care networks: a mixed-methods analysis of knowledge evaluation strategies and the success of informing family caregivers about dementia support services

**DOI:** 10.1186/s13033-016-0100-8

**Published:** 2016-10-12

**Authors:** Steffen Heinrich, Franziska Laporte Uribe, Markus Wübbeler, Wolfgang Hoffmann, Martina Roes

**Affiliations:** 1 German Center for Neurodegenerative Diseases (DZNE) - Site Witten, Stockumer Straße 12, 58453 Witten, Germany; 2 German Center for Neurodegenerative Diseases (DZNE) - Site Rostock, Ellernholzstraße 1-2, 17487 Greifswald, Germany; 3 Institute of Community Medicine, University of Greifswald, Ellernholzstraße 1-2, 17487 Greifswald, Germany

**Keywords:** Dementia, Networks, Support services, Home care, Knowledge management, Knowledge evaluation, Information

## Abstract

**Background:**

In general, most people with dementia living in the community are served by family caregivers at home. A similar situation is found in Germany. One primary goal of dementia care networks is to provide information on support services available to these caregiving relatives of people with dementia via knowledge management. The evaluation of knowledge management tools and processes for dementia care networks is relevant to their performance in successfully achieving information goals. One goal of this paper was the analysis of knowledge evaluation in dementia care networks, including potential barriers and facilitators, across Germany within the DemNet-D study. Additionally, the impact of highly formalized and less formalized knowledge management performed in dementia care networks was analyzed relative to family caregivers’ feelings of being informed about dementia support services.

**Methods:**

Qualitative data were collected through interviews with and semi-standardized questionnaires administered to key persons from 13 dementia care networks between 2013 and 2014. Quantitative data were collected using standardized questionnaires. A structured content analysis and a mixed-methods analysis were conducted.

**Results:**

The analyses indicated that the development of knowledge goals is important for a systematic knowledge evaluation process. Feedback from family caregivers was found to be beneficial for the target-oriented evaluation of dementia care network services. Surveys and special conferences, such as quality circles, were used in certain networks to solicit this feedback. Limited resources can hinder the development of formalized knowledge evaluation processes. More formalized knowledge management processes in dementia care networks can lead to a higher level of knowledge among family caregivers.

**Conclusions:**

The studied tools, processes and potential barriers related to knowledge evaluation contribute to the development and optimization of knowledge evaluation strategies for use in dementia care networks. Furthermore, the mixed-methods results indicate that highly formalized dementia care networks are especially successful in providing information to family members caring for people with dementia via knowledge management.

## Background

**Fig. 1:**
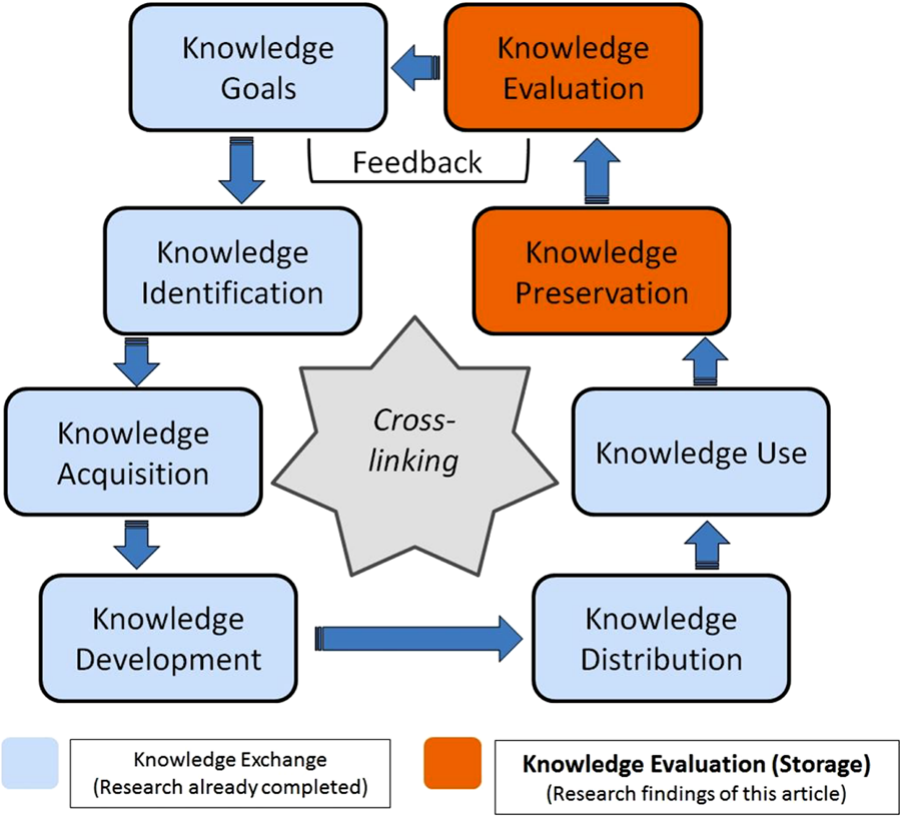
Knowledge management- and evaluation processes leaned on the knowledge management model by Probst et al. Probst [23]

Caring for people with dementia (PwD) at home is often associated with a considerable burden on family caregivers [1]. Although there are numerous dementia service stakeholders in Germany, a coordinated health care approach is often lacking; hence, the available support services are not as well aligned with the target groups (family caregivers and PwD) as they could be [1]. The establishment of organizations to support optimal collaboration between different dementia support stakeholders in the home care setting is seen as an essential goal by several countries [2]. In Germany, so-called dementia care networks (DCNs) have been founded in various regions to improve the coordination between dementia support stakeholders and caregivers for PwD living in the community [3–5]. These DCNs create links among health care professionals (e.g., social workers, physical therapists, nurses, and physicians) [6, 7]. Providing effective points of entry for information and support services for PwD and their caregivers is a primary goal of DCNs [8]. This goal is achieved through communication processes based on knowledge management (KM) for the development, utilization and exchange of knowledge. A systematic evaluation of these KM processes is thus essential for the successful achievement of this network goal [9]. Knowledge evaluation is an integral part of KM (Fig. 1). Furthermore, various aspects of KM are interconnected. For example, knowledge goals describe essential requirements for the structured creation of knowledge [10]. By evaluating these goals, it becomes possible to verify whether they have been achieved [10]. Furthermore, knowledge evaluation is one component of knowledge exchange processes. During such processes, the recipient must evaluate whether a given piece of knowledge is sufficiently relevant to be integrated and stored in a certain manner or should be rejected [11].

In the literature, numerous terms are used to refer to knowledge [12–14]. In this article, knowledge is defined as the target-oriented and reflective use of information [10]. This definition was selected because of its practice-relevant focus on current processes in the investigated DCNs. Knowledge evaluation is defined as the analysis of knowledge with the goal of optimizing existing KM tools and processes. Knowledge evaluation processes conducted by external persons or organizations are defined as “external evaluation”, whereas the evaluation tools and processes used within DCNs are classified as “internal evaluation”. Furthermore, in this report, “internal stakeholders” are defined as any contributing persons and/or organizations within a DCN. “External stakeholders” are defined as persons and/or organizations that are not part of the network but still play a relevant role in supporting PwD and their family caregivers. PwD and their caregivers are defined as “users” within the DCNs.

Thus far, no standard procedure has been developed to operationalize knowledge evaluation processes in DCNs in general, and very little is known about these processes within DCNs [15]. Furthermore, nothing is known of the potential factors affecting efforts to inform caregiving relatives about dementia support services via KM in DCNs.

The present paper reports the second phase of a program analyzing KM in differently structured DCNs. During the first phase, the KM practices in the investigated DCNs were analyzed with a focus on knowledge development and exchange, followed by a discussion about the related barriers and facilitators mentioned by the DCN stakeholders. These results have already been published in a previous article [4]. The current article focuses on knowledge evaluation (and storage) as the remaining aspects of KM. The previously researched aspects of KM as well as the remaining aspects analyzed in this paper are displayed in Fig. 1.

Specifically, this article focuses on the following aspects:

1. Description of formalized knowledge evaluation tools and processes used in DCNs, based on a KM model.
2. Description of attitudes, including barriers and facilitators, mentioned by involved DCN stakeholders with respect to knowledge evaluation.
3. Analysis of the correlation of KM in highly formalized and less formalized DCNs with the degree to which family caregivers feel informed about dementia support services (mixed-methods analysis).

This study is part of a larger study called DemNet-D, which has the purpose of evaluating the determining factors for the successful operation of DCNs with different areas of emphasis, for example, the impacts on caregiver burden or quality of life [3, 6, 7, 16, 17]. The overall DemNet-D study is funded by the German Federal Ministry of Health.

## Methods

### Qualitative data collection

Thirteen DCNs were included in this study. Three to eight key persons within every DCN were considered for the collection of qualitative data. In total, data were collected from 68 key DCN persons. The qualitative data presented in this article were acquired as part of the data collection described in the previously published article about KM in the investigated DCNs; that previous article also includes a table providing details about the key DCN persons’ characteristics [4].

Qualitative data were collected through single-person and group interviews using literature-based, pretested semi-standardized interview guidelines [4]. The emphasis of the group interviews was on selecting key people to reflect a variety of different professions to ensure that a wide range of perspectives were represented. Furthermore, these group discussions were used as a means of communicative validation of the findings from the round-one interviews [18]. The audio data from the two rounds of interviews were transcribed. Furthermore, a self-developed, semi-standardized questionnaire was developed and administered to the 13 DCN coordinators to extract the remaining details regarding the KM and knowledge evaluation processes used in the DCNs. By analyzing these data and merging them with the existing interview data from the two previously performed rounds of interviews, the information content reached saturation.

### Quantitative data collection

Data on the characteristics of the caregivers included in the mixed-methods analysis are displayed in Table 1.

**Table 1:** Caregivers characteristics (N = 565)^a^

Caregiver age in years (mean) [Range: min.–max.]	63.9 (SD ± 12.9) [24.0–93.0]
Caregiver gender (valid percentage, *n* = 555)	
Female	75.0 % (416)
Male	25.0 % (139)
Relationship with PwD (valid percentage, *n* = 559)^a^	
Spouse/partner	50.1 % (280)
Child	36.8 % (206)
Child-in-law	3.8 % (21)
Other	9.3 % (52)
Person with dementia age in years (mean)	79.7 (SD ± 8.4)
[Range: min.–max.]	[44.0–103.0]

*PwD* person with dementia

^a^Total numbers may vary due to missing values. Cases with missing values were excluded from the calculation of frequencies and means

The quantitative data used in the mixed-methods approach were based on items extracted from two standardized questionnaires used within the DemNet-D study [17, 19]. These items were drawn from the “Berlin Inventory of Caregivers” (BIZA-D) [20] and the “Instrument for Assessing Home-Based Care Arrangements for People with Dementia” (D-IVA) [21].

### Qualitative data analysis

A structured content analysis based on Mayring [22] was conducted with a focus on the qualitative interview and questionnaire material. The material was first subdivided into content paragraphs, which were then subdivided again into codes. Each code contained information about a specific piece of content consisting of a single word or a short passage. Among the investigated DCNs, different wording was often used to describe similar content. Therefore, the extracted codes were paraphrased. Based on the thematic structure of the Probst model [23], relevant content was allocated to specific categories. Figure 1 shows the scheme of the Probst model, which is widely accepted and used for the structuring of KM processes [24] and was also used for the structuring of the qualitative data in the previously published KM article [4].

The analysis was performed with the help of the software program MaxQDA 11 for qualitative analysis [25]. Based on this analysis, the formalized knowledge evaluation tools and processes used in the DCNs could be extracted. Furthermore, descriptions of the attitudes of the involved DCN stakeholders with respect to knowledge evaluation could be obtained. These data were then used as part of the subsequent mixed-methods analysis.

### Mixed-methods data analysis

A mixed-methods analysis was conducted to investigate the correlation of the KM in the DCNs with regard to the degree to which family caregivers feel informed about the available dementia support services. For this purpose, a mixed-methods triangulation design based on the data transformation model established by Creswell [26] was used. In this model, the data were transformed from one type (i.e., qualitative) into another (i.e., quantitative). Using this analysis model, it was possible to quantify the level of formalization in the DCNs based on the findings of the qualitative content analysis [27]. This process was necessary because the data on the family caregivers’ feelings of being informed about dementia support services were quantitatively structured.

With the aid of a score table, the DCNs were allocated into two groups according to their level of formalization. The score table (Table 2) was constructed using five primary content areas and eleven content items based on the KM model developed by Probst (Fig. 1).

**Table 2:** Scheme of the used mixed-methods tool

Data-label (cut-off scores)	KM area (based on Probst [8])	DCN-groups (persons/organizations)	Material-proof (+ − > formalized/− − > non formalized)	Result (+ = 1/− = 2)	
1.0–1.49 Highly formalized knowledge management 1.50–2.0 Less formalized knowledge management	Knowledge aims/identification	Internal stakeholders	E.g.: mission statements (~ +) or no formalization (~ −)	1 or 2	
	Knowledge development/acquisition	Internal stakeholders	E.g.: journal clubs (~ +) or no formalization (~ −)	1 or 2	+
		External stakeholders	E.g.: conferences (~ +) or no formalization (~ −)	1 or 2	+
	Knowledge distribution	Internal stakeholders	E.g.: IT-portals (~ +) or no formalization (~ −)	1 or 2	+
		External stakeholders	E.g.: informative materials (~ +) or no formalization (~ −)	1 or 2	+
		User	E.g.: press work (~ +) or no formalization (~ −)	1 or 2	+
	Knowledge use	Internal stakeholders	E.g.: guidelines (~ +) or no formalization (~ −)	1 or 2	+
	Knowledge evaluation	Internal stakeholders	E.g.: quality circles (~ +) or no formalization (~ −)	1 or 2	+
		External stakeholders	E.g.: research institutes (~ +) or no formalization (~ −)	1 or 2	+
		User	E.g.: feedback surveys (~ +) or no formalization (~ −)	1 or 2	+
	Knowledge storage	Internal stakeholders	E.g.: IT-libraries (~ +) or no formalization (~ −)	1 or 2	=
	*End-result*			*x/11 = 1.0–2.0*	

Based on the content items considered in the score table, cut-off scores were defined for the allocation of the 13 DCNs into two groups based on their level of formalization. Each content item was scored as either 1 (formalized) or 2 (unformalized). A total score of 22 points would indicate an unformalized status for all 11 content items considered by the tool, whereas a score of 11 points would indicate that a DCN was formalized with respect to every studied KM item. The arithmetic mean was calculated from the total score. DCNs with scores of 1.0–1.49 were defined as highly formalized, whereas the remaining DCNs, with scores from 1.50 to 2.0, were defined as less formalized. Most of the content items listed in the score table, with the exceptions of the “knowledge evaluation” and “knowledge storage” items, have already been analyzed (Fig. 1), and the results have been reported in the previously published KM paper [4].

The quantitative data on the family caregivers’ feelings of being informed were extracted from the D-IVA and BIZA-D. Three items were extracted from the D-IVA. Two of these items were rated on a binary scale (with values of “Yes” and “No”). The third item was based on a 4-point Likert scale ranging from 1 (very hard) to 4 (very easy). The fourth item was extracted from the BIZA-D and based on a 5-point Likert scale ranging from 0 (never) to 4 (always). The binary-scaled items were evaluated using the Pearson Chi Square test. The ordinal-scaled items were analyzed using the Mann–Whitney U Test because the sample data were not normally distributed. The findings were compared against the data from another project that focused only on PwD in the community [21] without considering DCNs. A statistical analysis was performed using the SPSS 19 software package [28].

## Results

### Knowledge evaluation tools and processes used in the DCNs

Knowledge evaluation processes performed by different stakeholders occur both within and outside of DCNs. In several cases, these processes appear to be performed with the assistance of unspecialized tools. The detailed results are displayed in Table 3.

**Table 3:** Knowledge evaluation and storage strategies in DCNs

Target area	Number of DCNs with formalized structures	Global DCN structures (number of notes by internal stakeholders [one count per network])	Processes/tools (number of notes by internal stakeholders [one count per network])
Internal DCN evaluation (internal stakeholders)	8/13	Working groups (7/8)	Performed by: General DCN evaluation in protocolled working groups (5/7) Evaluation of mission statement in quality circles (3/7) Literature-based knowledge evaluation in journal clubs (1/7)
		Feedback surveys (5/8)	Performed by: Network evaluation enquiry (4/5) Delphi census (1/5)
		QM-systems (5/8)	Used tools: Quality handbooks (4/5) KTQ (PDCA) (2/5) Balanced Scorecard (1/5)
Extraction of user feedback	7/13	IT systems (7/7)	Performed by: Homepage contact forms (6/7) Feedback hotline listed on homepage (1/7)
		Case management (7/7)	Performed by: Protocolled meetings between internal stakeholders and case managers (7/7) Case protocols of DCN users/external stakeholders [e.g., general practitioners] (5/7)
		Feedback surveys (5/7)	Used tools: Printed seminar feedback inquiries (5/5) Printed general feedback inquiries (3/5) Telephone inquiries (1/5)
		Conferences (4/7)	Performed by: Informative events with external stakeholders (3/4) Feedback forums between DCNs and users (2/4)
External performed evaluation	4/13	External research partners (4/4)	Performed by: Universities (3/4) Research institutes (1/4)
Information storage	13/13	Paper-based systems (13/13)	Used tools: File folders—general (13/13) Dementia network libraries for network Stakeholders (2/13) Dementia network libraries for network users (1/13)
		IT-systems (4/13)	Used tools: Internal literature databases (4/4) Internal IT-exchange forums (2/4)

Eight of the thirteen networks used formalized internal knowledge evaluation processes; these were primarily performed in working groups (7/8). Most of these processes occurred in general working groups, followed by KM-specific working groups known as quality circles, which were often used in the DCNs for the evaluation of mission statements. Mission statements are important for the establishment of knowledge goals [4]. Feedback surveys and quality management systems (e.g., balanced scorecard) were used in five of the DCNs for their knowledge evaluation processes. In four DCNs, external research partners performed knowledge evaluation processes. Three of these DCNs cooperated with universities for external knowledge evaluation, and one DCN collaborated with a private research organization.

Structures for the acquisition and extraction of user feedback had been developed in seven of the DCNs. The use of IT systems in combination with case management was common to all of these DCNs (7/7). Homepage contact forms were often used for IT-system-based feedback acquisition (6/7). Moreover, printed questionnaires were issued to users in many cases (5/7). One DCN conducted a telephone survey.

All 13 DCNs used common, paper-based folders to store information such as protocols or information material. IT-based information management systems were used in four DCNs (Table 3).

### Barriers, facilitators and attitudes of internal DCN stakeholders toward knowledge evaluation

The following quotations were each assigned a special code (based on Mayring). For example, *“This is a quotation”* (KR[code of the network]:EI[code of the interview]-421f.[content sector]). All quotations cited here were translated from German into English.

The interviewed internal stakeholders expressed different points of view with respect to knowledge evaluation in the DCNs. Furthermore, potential barriers were identified. Within the eight DCNs with formalized knowledge evaluation tools, all interviewed key persons acknowledged the importance of knowledge evaluation methods for assessing and illustrating the *success of specific DCN processes*. For example:

“We already use quality and knowledge evaluation tools in many areas of our network, and we wish to extend these processes to all fields. […] so that we get feedback: What suits and what does not.” (KR:EI-1617)

Furthermore, the interviewed stakeholders of six DCNs emphasized the importance of *receiving direct feedback* from DCN users to optimize services. For example:

“We are very excited about the success of this forum (user feedback forum; see Table 3). Everybody can equally discuss and spread new ideas. This is a fantastic basis for the further development of our network based on user wishes but also in general.” (AA:GD-151)

In two of the less formalized DCNs, internal stakeholders noted *concerns about developing formalized knowledge evaluation tools and processes*. In both networks, the stakeholders expressed the desire to avoid unneeded parallel structures:

“We (the stakeholders) are all using quality evaluation and feedback instruments (within their companies). We all know how they work, and we do it every day. We don’t need complex tools for knowledge evaluation in this network because we are all focused on direct and flexible communication.” (TK:EI-991)

Additionally, *barriers to formalized knowledge evaluation* in the DCNs were identified. In three of the less formalized DCNs, the interviewed internal stakeholders noted that they would prefer more formalized tools, but they noted a lack of personal resources for achieving this systematically. For example:

“We would like to have clear instruments for that (knowledge evaluation), but we don’t have them. […] We simply had no resources in our volunteer-based network until now.” (UK:EI-421f.)

*Furthermore, limited time* was noted by stakeholders of some of the highly formalized DCNs as a barrier to extending the existing knowledge evaluation tools and processes.

“We regard quality as providing opportunities for our network. Knowledge evaluation processes can improve our quality, but every new process for the systematic evaluation of our DCN work costs time, which is limited.” (PK:GD-479f.)

In addition to lack of time being a concern, *limited personal and professional resources* were noted as a barrier to the development of systematic knowledge evaluation processes.

“We have nobody to develop this in our network. We’re just learning by trial and error.” (AR:EI-100)

Another barrier observed in highly formalized DCNs was the *inappropriateness of certain evaluation instruments*. This situation led to the rejection of evaluation instruments in certain areas of the DCNs. Two examples are given below.

“Something we have tried and already given up is assessing the satisfaction of our users through static questionnaires. This heterogeneous group of people with different opinions and needs related to multiple support areas of our network could not be assessed using one single quantitatively based instrument. This approach didn’t work.” (AR:GD-549)

“We use a standardized questionnaire developed by the Alzheimer Society to evaluate the training of our users. The results are always perfect (laughing). That’s why I think it’s not selective enough. Who says that the seminar was stupid? Nobody.” (AA:GD-209)

### Correlation of the KM in the DCNs with regard to family caregivers’ knowledge of dementia support services (mixed-methods analysis)

Five DCNs (including n = 267 family caregivers) were assigned to the “highly formalized” group, and eight DCNs (including n = 298 family caregivers) were assigned to the “less formalized” group.

Relative to the level of DCN formalization, no significant differences were observed among the family caregivers’ need for dementia-specific information (Table 4—D-IVA 20.1). In both groups, most of the interviewed persons indicated that they needed dementia-specific information. Two of the three items (Table 4—D-IVA 20.2 and BIZA-D 4.13), which addressed problems in obtaining dementia support service information, revealed significant differences between the highly and less formalized DCNs. In the latter, significantly more problems in obtaining such information were encountered by the family caregivers in less formalized DCNs. The remaining item (Table 4—D-IVA Item 21) revealed no significant difference based on the level of formalization. Compared with caregivers for PwD who were not integrated into a DCN [21], both DCN groups (highly and less formalized) noted fewer problems in obtaining dementia-specific information with regard to all analyzed items (Table 4). Furthermore, in the sample presented by Kutzleben et al. the caregivers outside DCNs were found to have a higher need for dementia-specific information (97.6 %) compared with caregivers in highly (93.1 %) or less formalized (94.3 %) DCNs.

**Table 4:** Correlation of formalized KM processes in DCNs according to the family caregivers’ subjective degree of feeling informed - addendum comparison group

Instrument	Label CR* (n)	% CR* HF*^1^ (n)	% CR* LF*^1^ (n)	p value 95 % CI (x^2^)	% CR* total (n)	% CR* compar.*^2^ (n)
D-IVA (Item 20.1 + 20.2)	20.1 No need for dementia-specific information (558)^a^	6.9 (18)	5.7 (17)	*0.681*	6.4 (35)	2.4 (2)
	20.2 Need for dementia-specific information but no knowledge of how to obtain it (563)^a^	1.9 (5)	5.0 (15)	*0.048*	3.6 (20)	10.9 (9)

Instrument	Label	mean CR* HF*^1^ [SD] (n)	mean CR* LF*^1^ [SD] (n)	p value 95 % CI (U-Test)	mean CR* total [SD] (n)	mean CR* compar.*^2^ [SD] (n)
D-IVA (Item 21)	21. Appraisal of how difficult it is for a family caregiver of a PwD to obtain an overview about different types of dementia information and support services	2.43 [1.12] (245)	2.39 [1.17] (263)	*0.580*	2.41 [0.67] (508)^a^	2.29 [0.68] (72)
BIZA-D (Item 4.13)	4.13 Feelings about being hindered in obtaining information about support services for household care	0.89 [1.02] (242)	1.21 [1.27] (283)	*0.024*	1.05 [1.18] (525)^a^	No comparison data

* CR caring relatives

*^ 1^ *HF* highly formalized DCNs/*LF* less formalized DCNs

*^ 2^Comparison data from the VerAH-Dem project (Kutzleben [21] )

^a^ Total numbers may vary due to missing values. Cases with missing values were excluded from the calculation of frequencies and means

## Discussion

### Knowledge evaluation tools, processes and attitudes in the DCNs

One explanation for the frequent use of less clearly defined knowledge evaluation tools (e.g., general working groups) could be that unspecialized tools are more flexible than highly specialized knowledge evaluation tools. For example, general working groups or feedback surveys can be used for various processes and are not specially adapted for knowledge evaluation content [29, 30]. There are indications that a lack of personal resources and skills in DCNs is a frequent problem hindering the development of highly specialized knowledge evaluation tools and processes (UK:EI-421f./PK:GD-479f./AR:EI-100). Personal and time resources interact with each other, and negative impacts on knowledge evaluation can occur if there is a lack of these resources [31]. There must be sufficient financial resources to acquire professional staff with sufficient capacity to develop and oversee knowledge evaluation in DCNs [32]. These resources are equally important for the execution of knowledge distribution and exchange processes [4].

The process of extracting user feedback, as is done in certain DCNs, represents a generally important step for successful knowledge evaluation. By integrating user feedback, it is possible to clarify whether services are suitable or should be modified [33]. A formalized mission statement can be a helpful tool for the systematic analysis of DCN target achievement based on the merging of extracted user feedback with the knowledge goals expressed in the mission statement.

Informal knowledge evaluation processes were found to be favored in certain networks (AR:GD-549). Gupte [34] noted that an informal communication strategy can accelerate and simplify information flow. By contrast, the higher level of standardization of KM strategies offered by formalized processes could also be a potential advantage [34]. Certainly, uncertainties regarding the appropriateness of some formalized knowledge evaluation tools, particularly questionnaires, were observed in two DCNs (AR:GD-549/AA:GD-209). To avoid these barriers, tools should be tested with a focus on their validity and reliability to ensure that they are suitable for the specific knowledge evaluation processes for which they are intended to be used [35].

In the majority of the 13 DCNs (9/13), no specialized tools were used for the storage of evaluated information. However, the remaining four DCNs used IT-based information portals.

Users of these portals had the opportunity to receive, disseminate, modify and develop DCN information directly. The use of these tools can improve the dissemination of information and the evaluation of service quality because they allow all formalized DCN knowledge to be accessed in one centralized pool [36]. Therefore, the risk of creating niches or half-knowledge within fragmented stakeholder groups can be reduced by using a central information pool [37].

### Mixed-methods analysis of the degree to which caregivers feel informed

Among the analyzed items listed in Table 4, on item 20.1, only 25 out of 559 persons replied that they had no need for dementia-specific information. This statement underscores the importance of disseminating knowledge to PwD and their caregivers in the home care setting as a primary goal of DCNs [38]. Generally, the analysis indicated that several caregivers for PwD needed information on dementia support services, and most of them successfully obtained it through their DCNs. Compared with non-DCN users, users associated with DCNs experience more success in obtaining the information they require. However, the data indicate that DCNs with highly formalized KM strategies are even more successful than less formalized DCNs with respect to informing users, thus supporting the findings of Lemieux-Charles et al. [38] that highly formalized DCNs have more effective knowledge-sharing processes.

In another study, it was noted that large organizations in particular can benefit from clear formalized structures for coordinating and evaluating multiple concurrent processes [39]. However, a potential disadvantage of highly formalized structures is their higher demand for time resources, which are extremely limited in most DCNs. In Germany, formalization in the health care system is seen as an aspect of professionalization, and it is thus favored by most political stakeholders [40]. Nevertheless, small organizations, such as small DCNs, can occasionally operate more flexibly in response to customer needs by using relatively unformalized structures [41]. Hence, the optimal structure of a DCN depends on both its goals and its size.

A comparison of the data collected in this study with the data of Kutzleben et al. [21] clearly reveals that DCNs are successful with regard to the dissemination of knowledge. There are hints that DCNs can improve the dissemination of information concerning dementia and related support services for family caregivers of PwD.

## Limitations

In this study, it was not possible to gather qualitative information on the research topic from the perspective of PwD and caregiving relatives because of resource limitations. Moreover, it cannot be guaranteed that each relevant aspect of knowledge evaluation in DCNs could be extracted because of the high heterogeneity of the DCNs and the limited literature on this topic. However, the multiple rounds of data collection and the communicative validation of the material should limit the potential knowledge gaps. It is possible that other variables in addition to the level of DCN KM formalization may be correlated with the degree to which family caregivers feel informed. Nevertheless, all of the analyzed items support the hypothesis that a high level of formalization can yield improved processes for distributing knowledge to family caregivers. The data sample collected by Kutzleben et al., which was used for comparison, is small. Because of the sample size and the heterogeneity of the 13 DCNs, this study and its results must be regarded as explorative, thus limiting the generalizability of the findings to other DCNs. Furthermore, no standard definition of DCNs currently exists. Nevertheless, to the authors’ knowledge, this article presents the first dataset on knowledge evaluation in DCNs with this thematic scope and generates valuable findings focused on KM in DCNs.

## Conclusion

Most family caregivers noted a substantial need for obtaining dementia-specific information and reported successfully obtaining such information through their DCNs. The findings reported in this article indicate that in some of the DCNs evaluated in the DemNet-D-study, specially developed knowledge evaluation structures and processes are in use. Highly formalized DCNs appear to be even more effective in informing caregivers compared with less formalized DCNs; however, the investigated DCNs were generally successful in distributing knowledge to their users. IT-based information systems can be used for knowledge dissemination and evaluation processes by allowing information to be stored in an accessible, centralized location. Generally, DCNs seem to have the potential to increase the quality of information available and improve support for PwD and their caregivers through KM; however, insufficient personal and time resources can hinder KM processes in DCNs. This article can provide DCN stakeholders with information about the knowledge evaluation tools used in the studied DCNs. Further research should focus on the development of evidence-based KM tools to avoid knowledge gaps and support DCNs as expert structures in the field of dementia support. More information about the potential effects of KM tools in DCNs must be sought. Further analyses could, for example, address the effects of KM in DCNs on increasing the knowledge of the internal stakeholders as well as on professionalization and networking processes with respect to external stakeholders, such as general practitioners. In addition, cost-benefit calculations related to KM in DCNs would be very interesting and could generate value-based arguments for increasing funding for formalized KM structures and processes.

Some of the research findings on KM and knowledge evaluation that have been generated by the DemNet-D Project will be integrated into an already existing website that offers practice-focused recommendations for developing or founding new DCNs.^1^ Nevertheless, more systematic research on this topic is necessary to validate the findings presented in this article.

## Abbreviations

DCNs: dementia care networks
KM: knowledge management
PwD: people with dementia

## Footnotes

1
https://www.demenznetzwerke.de.
